# Distribution patterns of drug resistance *Mycobacterium tuberculosis* among HIV negative and positive tuberculosis patients in Western Kenya

**DOI:** 10.1186/s12879-021-06887-x

**Published:** 2021-11-22

**Authors:** Martin O. Ogwang, Mabel Imbuga, Caroline Ngugi, Lucy Mutharia, Gabriel Magoma, Lamec Diero

**Affiliations:** 1 School of Public Health, Jomo Kenyatta University of Agriculture and Technology, Nairobi, Kenya; 2 School of Biomedical Sciences, Jomo Kenyatta University of Agriculture and Technology, Nairobi, Kenya; 3 Department of Medicine, Moi University School of Medicine, Eldoret, Kenya; 4 Department of Cellular and Molecular Biology, University of Guelph, Guelph, ON Canada

## Abstract

**Introduction:**

Globally anti-tuberculosis drug resistance is one of the major challenges affecting control and prevention of tuberculosis. Kenya is ranked among 30 high burden TB countries globally. However, there is scanty information on second line antituberculosis drug resistance among tuberculosis patients. Therefore, this study aimed at determining *Mycobacterium tuberculosis* drug resistant strain distribution pattern in 10 counties of Western Kenya among HIV positive and negative patients.

**Method:**

A cross-sectional study was conducted in Western Kenya, which comprises 10 counties. A multistage sampling method was used where a single sub-county was randomly selected followed by sampling one high volume health facility from each sub-county. Consenting study subjects with at least two smear positive sputum at the time of enrolment were randomly selected. The collected sputum was decontaminated with *N*-acetyl-l-cysteine-sodium hydroxide (NALC-NaOH) and then stained with Ziehl Neelsen Stain before visualizing the presence of bacilli under microscope at ×100 magnification with oil immersion. Further, the identified bacilli were cultured and susceptibility test carried out using known first and second line antimycobacterial tuberculosis. HIV testing was carried out using Determine^®^ HIV-1/2 rapid test (Abbot Diagnostics, Maidenhead, United Kingdom). Those who had smear converted were dropped from the study. Finally, drug susceptibility pattern across the 10 counties of Western Kenya was evaluated.

**Results:**

Our study showed that *Mycobacterium tuberculosis* drug resistance among HIV negative and positive cases in Western Kenya was prevalent in all the 10 counties surveyed. Based on the drug susceptibility tests, 53.2% and 42.7% of the study samples were resistant to at least one antituberculosis drug among HIV negative and HIV positive patients respectively. The data analysis revealed that among the HIV-positive and HIV-negative patients, resistance to INH was predominant (28.5%, and 23.6%, respectively), followed by RIF (16.4% and 14.6% respectively). Second-line drug resistant strains identified among HIV negative patients included Ethionamide (0.3%), Gatifloxacin (0.3%), Amikacin (0.3%) and Capreomycin (0.3%). There was no second line drug monoresistance among HIV positive TB patients. Multi/poly drug resistance were noted among HIV-negative patients in, INH + AMK (0.7%), INH + PZA (1%), INH + GFX (0.7%, INH + ETO (0.7%, STY + ETO (1%), ETH + ETO (1.0%), INH + KAN (0.7%) and INH + CAP (0.7%) strains/cases at 95% confidence interval. Among HIV positive patients INH + GFX (1.1%), INH + ETO (0.4%) and INH + KAN (0.4%) strains of *M. tuberculosis* were identified with a confidence interval of 95%. Geographical distribution patterns analysis of *M. tuberculosis* drug polyresistant strains across the 10 counties were recorded. Among HIV TB patients, resistant strains were identified in Nyamira (INH + GFX, INH + KAN), Bungoma ((ETO + STY), Busia (ETH + ETO and STY + ETO) Homabay (RIF + AMK. ETO + ETH and ETO + STY), Kisumu (ETH + ETO and PZA + ETO) and in Kakamega, Kisii and Vihiga (INH + KAN and RIF + AMK). There was no *M. tuberculosis* polyresistant strain identified in Migori and Siaya counties. Among HIV positive TB patients, M. tuberculosis resistant strains were identified in three counties, Nyamira (INH + KAN) Homabay (INH + GFX and INH + AMK) and Kakamega (INH + GFX). There was no polyresistant *M. tuberculosis* strain identified in Migori, Bungoma, Kisii, Vihiga, Busia, Siaya and Kisumu Counties.

**Discussion:**

The distribution patterns of *M. tuberculosis* drug resistance among HIV negative and positive TB patients could be as a result of reported high prevalence of HIV in Western Kenya counties especially the area under study. Tuberculosis is one of the opportunistic diseases that have been shown to be the major cause of AIDS among HIV infected patients. Resent reports by National AIDS Control Council shows that Kisumu, Siaya, Homabay, Migori, Busia have the overall leading in HIV prevalence in Kenya. The low prevalence of drug resistant strains among HIV tuberculosis patients could be as a result of drug adherence attitude adopted by HIV patients, availability of continuous counselling and close follow up and notification by healthcare workers and community health volunteers.

**Conclusion:**

Drug resistant *M. tuberculosis* strains prevalence is still high among HIV negative and positive patients in Western Kenya with the most affected being HIV negative TB patients. It is therefore probable that the existing control measures are not adequate to control transmission of drug resistant strains. Further, miss diagnosis or delayed diagnosis of TB patients could be contributing to the emergence of *M. tuberculosis* drug polyresistant strains.

**Recommendation:**

Based on the result of this study, regular TB drug resistance surveillance should be conducted to ensure targeted interventions aimed at controlling increased transmission of the tuberculosis drug resistant strains among HIV/AIDS and HIV negative patients. There is also need for improved drug resistant infection control measures, timely and rapid diagnosis and enhanced and active screening strategies of tuberculosis among suspected TB patients need to be put in place. Further, studies using a larger patient cohort and from counties across the country would shed much needed insights on the true national prevalence of different variants of *M. tuberculosis* drug resistance.

## Introduction

Despite the global tuberculosis (TB) control interventions, TB is still a public health concern. Tuberculosis infection is currently ranked among the top three killer infectious diseases worldwide [1]. The current situation on TB infection and drug resistance is due to failure in prompt diagnosis, poor drug adherence, mismanagement of TB control programs, migration poverty, increased population and increased cases of TB/HIV cross infection [2].

Tuberculosis is the major killer of immunocompromised patients especially those infected with HIV [3]. Infection is almost exclusively transmitted airborne when an individual inhales live bacteria released in aerosolized microdroplets/droplet bioaerosols that are generated when an individual with active and advanced symptomatic pulmonary TB disease laughs, sneezes, coughs or is talking. Such aerosols can remain airborne and infectious for several hours, be carried in the air and accumulate in poorly ventilated environments. Factors that increase risk of transmission include: the bacilli load/cfu in the airborne bioaerosols, the concentration and distribution of bioaerosols in the environment; and prolonged exposure to bacillary-laden aerosols [4].

The emergence of drug resistance especially multidrug resistant tuberculosis (MDR-TB), defined as resistance to at least rifampicin and isoniazid, has been one of the impediments against tuberculosis management and prevention. As at 2016, the global estimated cases of multidrug resistant (MDR)-TB were 450,000 with 170,000 mortalities globally. In the same year 4.1% new cases were reported of which 19% were from previous treated cases. In the same year 8000 patients with extensively drug resistant (XDR)-TB cases were reported worldwide [5]. XDR-TB in addition of being MDR-TB, they are also resistant to any fluoroquinolones and to at least one of the injectable second-line drugs: kanamycin, capreomycin or amikacin. A more worrying trend has been the emergence of totally drug resistant tuberculosis (TDR)-TB *M. tuberculosis* strains resistant to all available anti-TB drugs [5].

In Africa there is incomplete data on drug resistance burden where only 24 of 47 (51%) of the countries having completed formal surveys in 2018 [6]. Considering this, modelled estimates of 2018 reported 92,829 combined drug resistant tuberculosis cases with about 42% occurring in Nigeria and South Africa [6]. In most of the cases identified 70% were not notified representing missed cases. The mortality rate of MDR-TB and XDR-TB in the continent is 21% and 43% respectively [6]. There has been a school of thought that, increases drug resistant tuberculosis in the continent is attributable to poor policy on the adoption of new diagnostic tools and lacking in implementation [6]. In Uganda, the national prevalence of MDR-TB is 2.3% while in Kampala the prevalence of this resistant strain has been reported to be 12.7% [7], however the prevalence among youths has been reported to be 1.69% [8]. In Tanzania, *M. tuberculosis* resistance prevalence to any of the anti-tuberculosis drugs is 8.3% while prevalence of MDR-TB is 1.1% [9]. In Kenya, studies carried out in 2019 reported that the prevalence of resistance to first and second line TB drugs has been reported to be 1.5% [10]. Kenya ranks 15 among the 22 high burden countries globally with national estimated HIV prevalence of between 5.6 and 6.3% [11].

Tubercle bacilli can survive in the dormant state as latent tuberculosis infection (LTBI) for years or decades. Approximately 5% of persons infected with LTBI will progress to active disease within 2 years of exposure to the bacillus. Another 5% will go on to develop active TB disease sometime later in their lifetime. Persons with immunocompromising conditions (e.g. HIV, chemotherapy, aged) are more likely to progress to active disease after infection or from reactivation of latent TB. For this latter groups, the risk of progressing to active disease is 10% per year [2].

In Kenya HIV prevalence is heterogenous across the 47 counties. The prevalence of HIV in Siaya, Kisumu and Homabay counties of Western Kenya accounts for 27% of all the patients on antiretroviral therapy. The prevalence of HIV in Western Kenya counties of Kisumu, Siaya and Homabay as per the 2018 estimates is at 15.3%, 17.5% and 19.6% respectively which is more than three times of the national prevalence of 4.9%. The prevalence in Migori, Busia, Kisii, Vihiga, Nyamira, Kakamega and Bungoma counties, HIV prevalence stands at 13%, 9.9%, 6.1%, 5.3%, 3.9%, 3.9% and 2.5% respectively [12]

Our study is the first to report tuberculosis drug resistant pattern among HIV negative and positive patients in Western Kenya which has been identified to be having high HIV prevalence rates for the last 20 years. The key strategy to prevention and management of all forms of drug resistance in TB is by early diagnosis. An understanding of drug resistant patterns will help in guiding policy formulation for better management of drug resistance tuberculosis.

## Materials and methods

### Study area

Western Kenya comprising of 10 counties (Bungoma, Busia, Siaya, Kakamega, Vihiga, Kisumu, Homabay, Migori, Kisii and Nyamira) was the study site. Sample processing was done at Moi University *Mycobacterium tuberculosis* Reference Laboratory (MRL, MUSOM) in Eldoret and Kenya National Tuberculosis Reference Laboratory (NTBRL) in Nairobi Kenya. The 10 counties of Western Kenya have been reported to be having the highest prevalence of HIV/AIDS infections in Kenya which is the major risk factor Tuberculosis which is an opportunistic disease. These counties account for 27% of patients on anti-retroviral treatment in Kenya. Additionally, these are border Counties bordering Uganda and Tanzania with very porous borders.

## Study design

### Study design and population

A cross sectional study design was used in this study.

### Ethical approval

The approval for the study was provided by Moi University Ethic Review Committee under the Reference number 0001519.

### Case definition

A case of tuberculosis disease was defined as a patient having two acid fast bacilli sputum positive test or a single positive *M. tuberculosis* culture. Mono resistant strains of *M. tuberculosis* are resistant to any single first line drug. MDR-TB resistant tuberculosis is resistant to Isoniazid with any other first line drug, and poly resistant here is defined as resistant to one second line drug and any first antituberculosis drugs.

### Sampling method

A multi stage sampling method was used to pick one subcounty and two high volume TB case health facilities in selected subcounty. This was followed by simple random sampling where samples were picked according to the proportionate prevalence of HIV and tuberculosis prevalence through balloting.

### Sample size determination

WHO 2011 for TB sample determination was employed [13],

$${\text{N}} = \frac{{{\text{n}}*{\text{z}}^{2} *{\text{p}}*(1 - {\text{p}})}}{{{\text{d}}^{2} *({\text{n}} - 1) + {\text{z}}^{2} *{\text{p}}*(1 - {\text{p}})}}$$

where N = Estimated sample size; n = number of smear positives = 141; P = Expected prevalence for smear positive samples from NTLP 2016 report (6%); Z = Corresponding confidence interval 95% (1.96); d = Precision or accuracy (0.02).

For cluster sampling Nt = N * d.e

where d.e: design effect, is a sample size multiplier taken as 2.

Therefore N = 110

$${\text{Nt}} = {11}0*{2} = {22}0$$

Added 20% to cater for contamination and loses

$$\left( {{22}0*0.{2}} \right) + {44} = {26}0$$

**Fig. 1:**
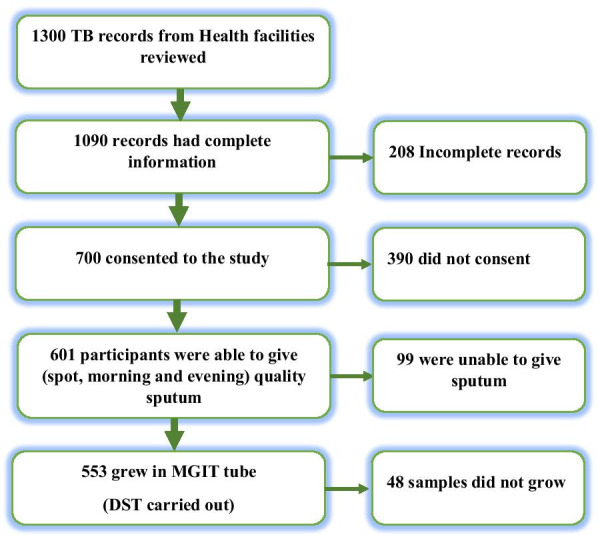
Flow diagram detailing how the study sample size was arrived at. TB study participant recruitment flow chart showing how the final sample size was arrived at

At the start of the study, 1300 were attending Chest clinics for TB therapy in selected health facilities in 10 counties of Western Kenya. Out of which 553 showed growth of Mycobacteria growth indicator tube. To reduce sampling bias, all the 553 samples were analyzed for drug susceptibility studies (Fig. 1).

### HIV testing

Provider initiated counselling and testing of HIV was carried out to determine the presence of HIV 1 and 2 antigens using determine^®^ HIV-1/2 rapid test (Abbot Diagnostics, Maidenhead, United Kingdom).

#### Collection and transportation of sputum

Three sputum specimens (spot, early morning, spot) were collected from participants suspected to have TB under the supervision of trained and competent medical staff. Upon consenting to the study, the patients were requested to generate a controlled deep cough to force expectorate from deep in the lungs, and spit into a sterile 50 mL blue cap tubes. The samples were refrigerated at 4 °C awaiting transportation in cool boxes to MRL, MUSOM weekly for analysis. Samples were processed within 7 days of collection in order to minimize loss of viability of the mycobacteria. Participants were also required to undergo phlebotomy for HIV testing. The blood was collected into Vacutainer (Brand STERILE interior EDTA (K3)) tubes and stored at − 20 °C waiting processing. Samples were transported in cool boxes to MRL, MUSOM, Eldoret, and processed within 2 weeks. Samples that needed further analysis with second line anti tuberculosis drugs were transported to National Tuberculosis Reference Laboratory for analysis.

### Identification of *Mycobacterium* bacilli from clinical samples

BACTEC MGIT 960 assay was used in determining mycobacterium strains from clinical samples. Briefly, the specimens were decontaminated and digested with an equal volume of 4% sodium hydroxide for 15 min and vortexing every 5 min, transferred into a 50 mL corning tube, centrifuged 3000 rpm for 15 min and the supernatant discarded. The pellets were washed twice by resuspending in 50 mL of phosphate-buffered saline (PBS pH 6.8) and centrifugation (3000 rpm, 15 min). The pellets were resuspended in 0.5 mL of PBS, inoculated into a MGIT culture tube and samples were incubated in the BACTEC MGIT 960 system until positivity was observed or up to 8 weeks for test-negative. Positive tubes were removed, confirmed for Acid fastness bacilli (AFB) using the Ziehl–Neelsen **(**ZN) protocol, and positive samples were then used for identification of *M. tuberculosis*-containing samples, drug susceptibility testing and for later storage of isolate.

### Identification *M. tuberculosis* from Mycobacterium other than tuberculosis (MOTT) strains

The Capilia TB Test Kit (TAUNS, Numazu, Japan) is a rapid, low technology method for differentiating *Mycobacterium tuberculosis* complex (MTBC) from Mycobacterium other than tuberculosis (MOTT). This immunochromatographic assay uses a monoclonal antibody to detect MPB64, one of the predominant proteins secreted by MTBC strains during culture [14–16]. The test is performed directly from positive ZN protocol cultures as previously described by [17]. Briefly, the Capilia TB assay was performed by placing 100 µL from a MGIT broth culture onto the specimen placement area of the Capilia TB cartridge, allowing a maximum of 15 min incubation and samples containing *M. tuberculosis* gave a purple–reddish color change in the test area containing the indicator. Negative samples with AFB-positive tests were considered as MOTTS and therefore eliminated from the study.

### Culture

*Mycobacterium tuberculosis* bacteria were cultured using the ABBL MGIT tube (Becton Dickinson) containing 7 mL modified Middlebrook 7H10 broth supplemented with OADC (oleic acid-dextrose-catalase) enrichment supplement [18] and an antibiotic cocktail of polymyxin B, amphotericin B, nalidixic acid, trimethoprim, and azlocillin were added [19]. After inoculation, the tubes were incubated at 37 °C. Readings were taken daily for the first 3 weeks and once a week thereafter for culture positivity until the end of 6 weeks using the BBL Micro MGIT system. All the positive tubes were further confirmed by ZN staining and a subculturing on blood agar plate and a Lowenstein–Jensen (LJ) agar media slant. The time to detection (TTD) of Mycobacteria were based on the date of the earliest instrumental indication of positivity.

### Drug susceptibility test

Drug susceptibility test was carried out as described by [20] with modification from Wedajo [21]. Briefly, for all antituberculosis drugs except PZA, Middlebrook (MB) 7H10 medium (Becton Dickinson and Company, Sparks, MD) supplemented with (OADC), pH 6.6; Becton Dickinson and Company) was used [22]. To prepare 500 mL medium solution, Middlebrook 7H10 powder (10 g) was added to 450 mL distilled water and mixed thoroughly. Subsequently, 2.5 mL glycerol (about 87% purity; BDH Laboratory Supplies, Poole, England) was added and the solution placed in a water bath at 100 °C to melt and completely dissolve the agar. The medium was sterilized for 10 min at 121 °C with 15 psi pressure and cooled to 50 °C in a water bath. A 50 mL of OADC, preheated to the same temperature, was added and 1 mL milliliter of agar and solidified then checked the final pH of the medium, which should amount to 6.6 ± 0.2. For the preparation of about 40 drug susceptibility (DST) plates for first line drugs, 2.5 L of Middlebrook 7H10 medium, supplemented with OADC (MB 7H10-OADC) was prepared. Additionally, conventional susceptibility testing was based on recommended critical concentrations (CC) [23]. In 23 bottles, each containing 2.5 mL of Middlebrook 7H10-OADC, dilutions of antituberculosis drugs in MB 7H10-OADC were prepared with the following final concentrations: 0.1, 0.2, 0.5, 1, and 2 µg/mL Isoniazid (INH-isonicotinic acid hydrazide; Sigma Chemical Co., St. Louis, MO); 0.1, 0.2, 0.5, 1, 2, and 5 µg/mL RIF (Rifampicin Sigma); 1, 2, 5, 10, and 20 µg/mL STY (streptomycin sulfate; Sigma); 1, 2, 5, 10, and 20 µg/mL ETH (ethambutol dihydrochloride; Sigma); 5 µg/mL amikacin (AMK; ICN Biomedicals, Inc., OH); 2.0 and 5.0 µg/mL ETH (Sigma); 1.0 µg/mL GFX (Sigma); 10 µg/mL CAP (Sigma) and 5.0 µg/mL KAN (Kanamycin Sigma) [17]. For quality control purposes, drug concentration other than critical concentration for some antituberculosis drugs were used. Growth with the following critical concentration of the test drugs defined resistance: INH 0.2 µg/mL, RIF 1.0 µg/mL, STY 2.0 µg/mL, ETH 5.0 µg/mL, ETO 5.0 µg/mL, GFX 1.0 µg/mL, CAP 10.0 µg/mL, and KAN 5.0 µg/mL. For AMK and PZA drugs with no WHO defined critical concentration for 7H10 medium at the time of the study, the previously suggested critical concentration based on wildtype distribution was used [20, 23, 24]. The antibiotic-containing media from the 23 bottles and from two bottles containing antibiotic-free medium (for the control wells) was transferred in 2.5-mL amounts into 25-well plates (Greiner Bio-One, Alphen a/d Rijn, The Netherlands). The culture media was dispensed using a 25-channel dispenser fitted with an electronic pump (RIVM). To solidify the medium, the plates are left at room temperature for approximately 1 h. The plates are stored at 4 °C until use.

For PZA testing, MB 7H10-OADC was adjusted to pH of 5.7 ± 0.15 using 3 M HCl. PZA was added to a final concentration of 10, 20, 50, and 100 µg/mL [20].

### Inoculation and incubation of the DST plates

The *Mycobacterium* isolates from the MGIT tubes to be tested were suspended by inoculating using a small loop of bacterial culture in a 40 mL sterile water in unbreakable plastic bottles (Balis, Boven-Leeuwen, The Netherlands). The suspensions were homogenized by shaking with glass beads with diameter 1.4 mm for 20 min at 350 rpm in a closed biosafety bucket (Lab-Line model 4626-ICE; Beun-de Ronde, Abcoude, The Nether lands). To allow sedimentation of coarse-grained particles, the suspensions are incubated at room temperature for 10 min. The turbidity of the supernatant was adjusted to a stock solution with a McFarland standard of 1 to obtain a density of 2 × 10^5^ to 10 × 10^5^ CFU/mL. Sterile water was added to dilute high turbidity suspensions.

The 25-well DST plates were inoculated with 10 µL of the suspensions to 24 wells using an automated pipette (Socorex, Omnilabo, Breda, The Netherlands). The final well, one of the control wells in each plate (contained antibiotic-free medium), was inoculated with a 1/100 dilution of the mycobacterial suspension, allowing the quantification/counting of cfu bacteria in the inoculum after incubation. The plates were incubated at 35.5 °C in a CO_2_ incubator with a water reservoir to prevent drying of the plates.

### Reading of DST plates

*Mycobacterium tuberculosis* growth were checked after 6, 12, and 19 days. Plate reading were carried out when bacterial colonies were clearly visible on the two control wells without antituberculosis (anti-TB) drugs. The MIC, the lowest concentration of antituberculosis drugs that inhibits more than 99% of target bacterial growth, was determined by a comparison to cfu on the control well. The interpretation of the MIC readings, i.e., the decision as to whether a strain is susceptible or resistant to a certain drug, is based on the critical concentration. Growth at the critical concentration is reported as resistant, and growth at higher concentrations of drug is considered resistant.

### PZA drug susceptibility testing

PZA, a nicotinamide analogue, is reportedly more active in vivo than the in vitro susceptibility studies indicate. Presumably, the main metabolite, pyrazinoic acid, which reaches even higher plasma levels than does PZA after an oral dose of PZA, contributes significantly to the activity of the drug in vivo.

Since the optimum activity of PZA in vitro is expressed under acid conditions, the culture media was adjusted to pHs 5.0 to 5.5. At this pH range, the MICs on solid media for susceptible *M. tuberculosis* strains is usually 25 to 100 µg/mL; at a neutral pH, these values are at least 10 times higher. The PZA susceptibility testing media used in this study was based on the work of [20] where bacteria were cultured on pH 5.5 7H10 agar, modified with a specific ratio of buffering phosphate salts.

#### Quality control

Quality assurance of DST is ensured at four different levels. Each 25-well DST plates contained two control wells one with 1 µg/mL PAS and one with 5 µg/mL AMK; both wells serve as controls to check the purity and identification of each of the isolates. In general, *M. tuberculosis* complex isolates do not grow on medium with these drugs. For each series of *M. tuberculosis* isolates inoculated on DST plates, one *Mycobacterium gordonae* strain, one *Mycobacterium avium* strain, and three *M. tuberculosis* control strains with known MICs were included as internal controls in each experiment.

### Data analysis

SPSS version 21 was used in data management. Descriptive analysis was done to illustrate proportions on the social demographic variables, drug sensitive and resistance TB among HIV negative and positive participants and frequencies and percentages. Significant differences in proportion between different groups were assessed Pearson Chi square test with P ≤ 0.05 considered significant.

## Results

### Social demographic characteristics of the study population

Out of the 1300 TB patient records which were randomly determined from different health facilities, 1090 (83.8%) had complete record entries out of which 700 (53.8%). Out of those who consented to participating in the study, 601 (85.8%) were able to produce deep spot, morning and evening sputum for analysis. From those who consented to participate in the study and who were able to provide quality sputum, 553 (79%) of the sputum samples were positive for *M. tuberculosis* bacilli culture and therefore were subjected to drug susceptibility test (Fig. 1).

This study captured participants aged 18 years and above. The most affected were in the age bracket of 25–30 years representing 33% of the sample size. Of the total sample size, 50.8% were HIV negative while 49.2% were HIV positive. Majority of the sampled participants (62.4%) were males while the rest were females. Most participants sampled were primary school leavers (44%), 26% with informal education, 23% secondary school leavers while only 7% of the participants. The most affected social economic cadre was self-employed persons (37%) followed by those with informal employment (20%), the affected unemployed were 16% while 15% were employed with 10% still in school. Participants with monthly income in the bracket of 10,000–19,999 were 30% with the least being those with monthly income of Ksh. 50,000 and above (Table 1).

**Table 1:** Social demographic characteristics of the study population (N = 553)

Variable	Status	Frequency	Percentage
Age	18–24	145	26.1
	25–30	183	33.1
	31–36	107	19.3
	37–42	47	8.5
	43–48	35	6.3
	49–54	18	3.3
	55–60	12	2.2
	61+	6	1.1
HIV status	Positive	267	48.3
	Negative	286	51.7
Sex	Female	208	37.6
	Male	345	62.4
Education	No formal	144	26
	Primary	243	44
	Secondary	127	23
	Tertiary	39	7
Occupation	Employed	83	15
	Self employed	218	39
	Informal employment	128	23
	Unemployed	103	16.6
	Student	10	1.8
	Other	11	2
Monthly income	Less than Ksh. 1000	105	19
	Ksh. 1000–9, 999	160	28.9
	Ksh. 10,000–19,999	174	31.5
	Ksh. 20,000–29,999	48	8.7
	Ksh. 30,000–39,999	34	6.1
	Ksh. 40,000–49,999	18	3.3
	Ksh. 50,000+	14	2.5
Size of the house	Single room	122	22.1
	Bedsitter	11	2
	One bedroom	207	37.4
	Two bedroom	191	34.5
	Other	22	4
Cigarate smocking	Yes	132	23.9
	No	421	76.1
Alcohol use	Yes	151	27.3
	No	402	72.7
Cross boarder travels	Yes	211	38.2
	No	342	61.8

In terms of housing, those living in single bedroomed house were the most sampled representing 44.6%, while those living in two bedrooms were 20.4%, those living in bedsitters were 17%, and those in single rooms were 16%, with 2% of unknown abode.

Also, among the sampled, 75% were non Cigarate smokers while 25% were smokers; 51.5% did were drink alcohol while 48.4% were alcohol users.

Majority of participants had only travelled locally (69.2%), 26.6% had traveled within East African countries but only 4% and 0.2% had traveled within African and outside Africa respectively (Table 1).

### Patterns of resistance to first line antituberculosis drug in Western Kenya

From the drug susceptibility study, 42.7% and 53.2% of the study samples were resistant at least one antituberculosis drug among HIV negative participants and HIV positive patients, respectively. Screening for any form of resistance indicates that isoniazid (INH or H) resistance was the most frequent among HIV negative and positive population with a frequency 28.5% and 23.6% respectively at 95% confidence interval. Rifampicin (RIF or R) resistance was 16.4 and 14.6 among HIV negative and positive participant population, respectively. Ethambutol (ETH or E), Streptomycin (STY or S) and pyrazinamide (PZA or P) resistance frequency among HIV negative TB patients were 4.5%, 4.2%, and 1.7%, respectively while in HIV positive TB cohort the proportion of these drug frequency was 5.6%, 2.2% and 2.6% respectively. Ethionamide (ETH), Gatifloxacin (GFX), Amikacin (AMK) and Capreomycin (CAP) resistance proportions among HIV negative were 0.3%, 0.3%, 0.3% and 0.3% respectively, while in HIV positive TB patients no resistance to these was reported.

Isoniazid drug monoresistant was the highest among HIV negative and positive TB patients with proportions indicating 18.2% and 12.9% drug mono resistance respectively. The proportion for RIF, EMB, STY and PZA monoresistance among HIV negative TB patients were 9.4%, 1.0%, 3.1% and 3.1% respectively while among HIV positive TB patients the proportions were 9.0%, 2.6%, 1.9% and 1.9% respectively at 95% CI (Table 2).

**Table 2:** Patterns of resistance to first and second line antituberculosis drug in Western Kenya

Item	HIV negative TB patients (N = 286)			HIV positive TB patients (N = 267)				Total (N = 553)		
	No	%	95% CI	No	%		95% CI	No	%	95% CI
No. sensitive to all	164	57.3	51.7–63.3	125	46.8		40.4–53.9	286	51.7	47.7–56.1
Drug resistant strains	122	42.7	36.7–48.3	142	53.2		46.1–59.6	267	48.3	43.9–52.3
Any resistance										
Isoniazid (H)	81	28.5	23–34	63	23.6		18.4–29.2	144	26	22.6–29.5
Rifampicin (R)	47	16.4	12–20.7	39	14.6		10.1–19.1	86	15.6	12.6–19
Ethambutol (E)	13	4.5	2.1–7.0	15	5.6		3.0–8.7	28	5.1	3.3–6.7
Streptomycin (S)	12	4.2	2.1–6.6	6	2.2		0.7–4.1	18	3.3	2.0–4.9
Pyrazinamide	5	1.7	0.3–3.5	7	2.6		0.7–4.5	12	2.2	1.1–3.5
Ethionamide	1	0.3	0.0–1.4	0	0		–	1	0.2	0.0–0.5
Gatifloxacin	1	0.3	0.0–1.0	0	0		–	1	0.2	0.0–0.5
Amikacin	1	0.3	0.0–1.0	0	0		–	1	0.2	0.0–0.5
Capreomycin	1	0.3	0.0–1.0	0	0		–	1	0.2	0.0–0.5
Mono-resistant TB										
Isoniazid (H)	52	18.2	14.3–22.7	34	12.7		6–12.4	86	15.6	12.7–18.7
Rifampicin (R)	27	9.4	5.6–12.9	24	9.0		9–16.5	51	9.2	6.9–11.8
Ethambutol (E)	3	1.0	0.0–2.4	7	2.6		0.4–4.9	10	1.8	0.7–2.9
Streptomycin (S)	9	3.1	1.4–5.3	5	1.9		0.4–3.7	14	2.5	1.4–4.0
Pyrazinamide	9	3.1	1.0–5.6	5	1.9		0.4–3.7	14	2.5	1.3–4.0
Multidrug resistant TB (MDR-TB)										
H + R	16	5.6	3.1–8.1	17	6.4		3.4–9.7	33	6	4.2–8.1
H + R + E	12	4.2	2.1–6.6	13	4.9		2.2–7.5	25	4.5	2.9–6.5
H + R + S	3	1.0	0.0–2.1	0	0		–	3	0.5	0.0–1.3
H + R + E + S	0		–	2	0.7		0.0–1.9	2	0.4	0.0–0.9
Polyresistance										
R + PZA	3	1	0.0–2.1	3	1.1		0.0–2.6	6	1.1	0.4–2.0
R + Amk	2	0.7	0.0–1.7	–	–		–	2	0.4	0.0–0.9
H + PZA	3	1	0.0–2.4	–	–		–	3	0.5	0.0–1.3
H + Gfx	2	0.7	0.0–1.7	3	1.1		0.0–2.6	5	0.9	0.0–1.3
H + Eto	2	0.7	0.0–1.7	1	0.4		0.0–1.5	3	0.5	0.0–1.1
STY + Eto	1	1	0.0–1.4	–	–		–	1	0.2	0.0–0.7
ETH + Eto	3	1	0.0–2.4	–	–		–	3	0.5	0.0–1.3
ETH + PZA	2	0.7	0.0–1.7	–	–		–	2	0.4	0.0–0.9
H + Kn	2	0.7	0.0–1.7	1	0.4		0.0–1.1	3	0.5	0.0–1.3
H + Cm	1	0.3	0.0–1.0	–	–		–	1	0.2	0.0–0.7
H + Amk	–	–	–	3	1.1		0.0–2.6	3	0.5	0.0–1.3

Isoniazid and Rifampicin (H + R) was the most observed multidrug resistance in both HIV negative and positive TB patient cohorts with a proportion of 5.6% and 6.4% respectively. While Isoniazid + Rifampicin + Ethambutol multidrug resistance MDR drug combination was identified at a proportion of 4.2% and 4.9% respectively among HIV negative and positive TB patients. Further, drug susceptibility results reported no H + R + S, MDR combination phenotype among HIV positive TB patients while a proportion of 1% was identified among HIV negative patients. However, 2% of H + R + E + S multidrug resistance phenotypes were identified among HIV positive patients but no sample of this phenotype was identified among HIV negative TB patients (Table 2).

Finally, antituberculosis drug poly resistance was also observed among HIV negative and positive TB patient participants. For HIV negative, RIF + PZA, INH + Amk, INH + PZA, INH + GFX, INH + ETH, STY + ETH, EMB + ETH, EMB + PZA, INH + KAN and INH + CAP antituberculosis drug polyresistant proportions were reported as 1%, 0.7%, 1%, 0.7%, 0.7%, 1%, 1%, 0.7%, 0.7% and 0.3% respectively. While among HIV positive TB patient participants, only RIF + PZA, INH + GFX, INH + ETH and INH + KAN polyresistance phenotypes were with proportions of 1.1%, 1.1%, 0.4%, and 0.4% respectively (Table 3).

**Table 3:** Geographical distribution patterns of *M. tuberculosis* drug mono resistance strains isolated from HIV negative and positive TB patients in Western Kenya

County	HIV negative						HIV positive					
	RIF R	INH R	STY R	ETH R	PZA R	Total	RIF R	INH R	STY R	ETH R	PZA R	Total
Nyamira	**2 (0.7%)**	0 (0.0%)	**1 (0.3%)**	0 (0.0%)	0 (0.0%)	**3 (1.0%)**	0 (0.0%)	**2 (0.7%)**	0 (0.0%)	**2 (0.7%)**	0 (0.0%)	**4 (1.5%)**
Bungoma	**1 (0.3%)**	**3 (1.0%)**	**2 (0.7%)**	**2 (0.7%)**	**1 (0.3%)**	**9 (3.1%)**	**1 (0.4%)**	**1 (0.4%)**	0 (0.0%)	0 (0.0%)	0 (0.0%)	**2 (0.7%)**
Busia	**2 (3.5%)**	**8 (2.8%)**	**1 (0.3%)**	0 (0.0%)	**3 (1.0%)**	**14 (4.9%)**	**1 (0.4%)**	**7 (2.6%)**	**1 (0.4%)**	0 (0.0%)	**2 (0.7%)**	**11 (4.1%)**
Homabay	**10 (3.5%)**	**9 (3.1%)**	**1 (0.3%)**	0 (0.0%)	**1 (0.3%)**	**21 (7.3%)**	**1 (0.4%)**	**7 (2.6%)**	**1 (0.4%)**	0 (0.0%)	**1 (0.4%)**	**10 (3.7%)**
Kakamega	**1 (0.3%)**	**4 (1.4%)**	**0 (0.0%)**	0 (0.0%)	0 (0.0%)	**5 (1.7%)**	**3 (1.1%)**	**2 (0.7%)**	0 (0.0%)	0 (0.0%)	**1 (0.4%)**	**6 (2.2%)**
Kisumu	**4 (1.4%)**	**7 (2.4%)**	**4 (1.4%)**	**1 (0.3%)**	**2 (0.7%)**	**18 (6.3%)**	**7 (2.6%)**	**3 (1.1%)**	**2 (0.7%)**	**3 (1.1%)**	**1 (0.4%)**	**15 (5.6%)**
Migori	**3 (1.0%)**	**7 (2.4%)**	**1 (0.3%)**	0 (0.0%)	**1 (0.3%)**	**12 (4.2%)**	**4 (1.5%)**	**5 (1.9%)**	0 (0.0%)	**1 (0.4%)**	0 (0.0%)	**10 (3.7%)**
Kisii	**2 (0.7%)**	**4 (1.4%)**	0 (0.0%)	**1 (0.3%)**	0 (0.0%)	**7 (2.4%)**	**2 (0.7%)**	**1 (0.4%)**	0 (0.0%)	0 (0.0%)	0 (0.0%)	**3 (1.1%)**
Vihiga	**1 (1.0%)**	**5 (1.7%)**	0 (0.0%)	**1 (0.3%)**	**1 (0.3%)**	**8 (2.8%)**	**1 (1.4%)**	**3 (1.1%)**	**1 (0.4%)**	**1 (0.4%)**	0 (0.0%)	**6 (2.2%)**
Siaya	**1 (0.3%)**	**5 (1.7%)**	**1 (0.3%)**	0 (0.0%)	**1 (0.3%)**	**8 (2.8%)**	**4 (1.5%)**	**3 (1.1%)**	0 (0.0%)	0 (0.0%)	0 (0.0%)	**7 (2.6%)**
Total	**27 (9.4%)**	**52 (18.2%)**	**11 (3.8%)**	5 (1.7%)	**10 (3.5%)**	**105 (36.7%)**	**24 (9.0%)**	**34 (12.7%)**	**5 (1.9%)**	**7 (2.6%)**	**4 (1.5%)**	**74 (27.7%)**

The bold was just used to track entries

### Geographical distribution patterns of *M. tuberculosis* drug mono resistance strains isolated from HIV negative and positive TB patients in Western Kenya

*Mycobacterium tuberculosis* mono resistance was detected in all the 10 counties of Western Kenya among HIV positive TB with RIF and INH being detected in almost all counties. RIF, INH, STY, ETH and PZA mono resistance was detected in Bungoma and Kisumu counties while in Busia, Homabay, Migori, Kisii, Siaya and Vihiga reported RIF, INH, STY and PZA mono resistance. In Nyamira, Rifampicin and Streptomycin mono resistant phenotypes were detected while in Kakamega, Rifampicin and Isoniazid mono resistant bacilli were reported (Table 3).

Among HIV positive TB patients who participated in the study, just like in HIV negative cohort, RIF and INH were detected in almost all the 10 counties of Western Kenya apart from Nyamira where no RIF resistance was detected. In Kisumu County five mono resistant phenotypes were detected which were RIF, INH, STY, ETH and PZA. On the other hand, RIF, INH, STY and PZA mono resistant phenotypes were detected in Busia and Homabay counties. In Bungoma, Kisii and Siaya counties, RIF and INH monoresistant phenotypes were detected. Additionally, RIF, INH, STY and ETH mono resistant phenotypes were reported in Vihiga counties while Migori county reported three phenotypes which were RIF, INH and ETH were identified. From the study, Kakamega county reported RIF, INH and PZA mono resistant phenotypes while in Nyamira county RIF and INH mono resistant *M. tuberculosis* phenotypes were observed (Table 3).

### Geographical distribution patterns of *M. tuberculosis* MDR strains isolated from HIV negative and positive TB patients in Western Kenya

In HIV positive TB cohort, the most predominant MDR phenotypes was HR which was reported in Nyamira, Busia, Homabay, Kakamega, Kisumu, Migori, Kisii and Siaya counties. On the other hand, HRE phenotypes were reported in Busia, Homabay. Kisumu, Kisii, Vihiga and Siaya counties. Multidrug resistant tuberculosis of the phenotype HRS was detected in Homabay, Kisumu and Siaya counties. However, in Bungoma County there was no MDR phenotype that was reported among HIV negative TB patients (Table 4).

**Table 4:** Geographical distribution patterns of *M. tuberculosis* MDR strains isolated from HIV negative and positive TB patients in Western Kenya

County	HIV negative					HIV positive				
	HR	HRE	HRS	HRES	Total	HR	HRE	HRS	HRES	Total
Nyamira	**1 (0.3%)**	0 (0.0%)	0 (0.0%)	0 (0.0%)	**1 (0.3%)**	**2 (0.7%)**	**1 (0.4%)**	0 (0.0%)	0 (0.0%)	**3 (1.1%)**
Bungoma	0 (0.0%)	0 (0.0%)	0 (0.0%)	0 (0.0%)	**0 (0.0%)**	**1 (0.4%)**	**1 (0.4%)**	0 (0.0%)	0 (0.0%)	**2 (0.7%)**
Busia	**2 (0.7%)**	**3 (1.0%)**	0 (0.0%)	0 (0.0%)	**5 (1.7%)**	**1 (0.4%)**	**1 (0.4%)**	0 (0.0%)	0 (0.0%)	**2 (0.7%)**
Homabay	**5 (1.7%)**	**4 (1.4%)**	**1 (0.3%)**	0 (0.0%)	**10 (3.5%)**	**1 (0.4%)**	0 (0.0%)	0 (0.0%)	0 (0.0%)	**1 (0.4%)**
Kakamega	**3 (1.0%)**	**0 (0.0%)**	0 (0.0%)	0 (0.0%)	**3 (1.0%)**	**3 (1.1%)**	**2 (0.7%)**	0 (0.0%)	0 (0.0%)	**5 (1.9%)**
Kisumu	**2 (0.7%)**	**1 (0.3%)**	**1 (0.3%)**	0 (0.0%)	**4 (1.4%)**	**1 (0.4%)**	**2 (0.7%)**	0 (0.0%)	0 (0.0%)	**3 (1.1%)**
Migori	**1 (0.3%)**	0 (0.0%)	0 (0.0%)	0 (0.0%)	**1 (0.3%)**	**3 (1.1%)**	**3 (1.1%)**	0 (0.0%)	**1 (0.4%)**	**7 (2.6%)**
Kisii	**1 (0.3%)**	**1 (0.3%)**	0 (0.0%)	0 (0.0%)	**2 (0.7%)**	**1 (0.4)**	**2 (0.7%)**	0 (0.0%)	0 (0.0%)	**3 (1.1%)**
Vihiga	0 (0.0%)	**1 (0.3%)**	0 (0.0%)	0 (0.0%)	**1 (0.3%)**	**2 (0.7%)**	0 (0.0%)	0 (0.0%)	**1 (0.4%)**	**3 (1.1%)**
Siaya	**1 (0.3%)**	**2 (0.7%)**	**1 (0.3%)**	0 (0.0%)	**4 (1.4%)**	**2 (0.7%)**	**1 (0.4%)**	0 (0.0%)	0 (0.0%)	**3 (1.1%)**
Total	**16 (5.6%)**	**12 (4.2%)**	**3 (1.0%)**	**0 (0.0%)**	**31 (10.8%)**	**17 (5.9%)**	**13 (4.5%)**	**0 (0.0%)**	**2 (0.7%)**	**32 (12.0%)**

The bold was just used to track entries

Among HIV positive TB patients again the most predominant MDR TB phenotype reported was HR which was detected in Nyamira, Bungoma, Busia, Homabay, Kisumu, Migori, Kisii, Vihiga and Siaya counties. This was followed by HRE phenotype that were distributed in Nyamira, Bungoma, Busia, Kisumu, Migori, Kisii and Siaya counties. Vihiga and Migori counties were the only counties that reported HRES MDR phenotype among HIV positive patients (Table 4).

### Geographical distribution patterns of *M. tuberculosis* drug polyresistant strains isolated from HIV negative and positive TB patients in Western Kenya

Several poly resistant profiles were reported among HIV negative TB patients in Western Kenya. In Nyamira INH + GFX and INH + KAN were detected, in Bungoma, RIF + PZA and ETH + STY phenotypes were detected. In Busia county EMB + ETH and ETH + STY strains were identified. Homabay county had three strains of M. tuberculosis polyresistant were reported which included RIF + AMK, EMB + ETH and ETH + STY while in Kisumu County, EMB + ETH and ETH + PZA polyresistant phenotypes were detected. In Kakamega, Kisii and Vihiga counties INH + KAN and RIF + AMK phenotypes were reported respectively. However, there was no poly resistant phenotype identified in Migori and Siaya counties among HIV negative TB patients (Table 5).

**Table 5:** Distribution patterns of *M. tuberculosis* drug polyresistant strains isolated from HIV positive and Negative TB patients in Western Kenya

County	Polyresistant drug combination among HIV positive TB patients					Polyresistant drug combination among HIV negative TB patients					
	INH + GFX	INH + AMK	INH + ETH	INH + KAN	Total	RIF + AMK	INH + GFX	INH + KAN	ETH + ETH	ETH + STY	Total
Nyamira	0 (0.0%)	0 (0.0%)	0 (0.0%)	**1 (0.4%)**	**1 (0.4%)**	0 (0.0%)	**1 (0.3%)**	**1 (0.3%)**	0 (0.0%)	0 (0.0%)	**4 (1.4%)**
Bungoma	0 (0.0%)	0 (0.0%)	0 (0.0%)	0 (0.0%)	**0 (0.0%)**	0 (0.0%)	**0 **(0.0%)	0 (0.0%)	0 (0.0%)	**1 (0.3%)**	**2 (0.7%)**
Busia	0 (0.0%)	0 (0.0%)	0 (0.0%)	0 (0.0%)	**1 (0.4%)**	0 (0.0%)	0 (0.0%)	0 (0.0%)	**1 (0.3%)**	**1 (0.3%)**	**3 (1.0%)**
Homabay	**1 (0.4%)**	**2 (0.7%)**	0 (0.0%)	0 (0.0%)	**3 (1.1%)**	**1 (0.3%)**	0 (0.0%)	0 (0.0%)	**1 (0.3%)**	**1 (0.3%)**	**5 (1.7%)**
Kakamega	**1 (0.4%)**	0 (0.0%)	0 (0.0%)	0 (0.0%)	**1 (0.4%)**	**0 **(0.0%)	**0 **(0.0%)	0 (0.0%)	0 (0.0%)	0 (0.0%)	**1 (0.3%)**
Kisumu	0 (0.0%)	0 (0.0%)	0 (0.0%)	0 (0.0%)	**1 (0.4%)**	0 (0.0%)	0 (0.0%)	0 (0.0%)	**1 (0.3%)**	0 (0.0%)	**3 (1.0%)**
Migori	0 (0.0%)	**1 (0.4%)**	**1 (0.4%)**	0 (0.0%)	**2 (0.7%)**	0 (0.0%)	0 (0.0%)	0 (0.0%)	0 (0.0%)	0 (0.0%)	**0 (0.0)**
Kisii	0 (0.0%)	0 (0.0%)	0 (0.0%)	0 (0.0%)	**0 (0.0%)**	0 (0.0%)	0 (0.0%)	**1 (0.3%)**	0 (0.0%)	0 (0.0%)	**1 (0.3%)**
Vihiga	0 (0.0%)	0 (0.0%)	0 (0.0%)	0 (0.0%)	**0 (0.0%)**	**1 (0.3%)**	0 (0.0%)	0 (0.0%)	0 (0.0%)	0 (0.0%)	**1 (0.3%)**
Siaya	0 (0.0%)	0 (0.0%)	0 (0.0%)	0 (0.0%)	**1 (0.4%)**	0 (0.0%)	0 (0.0%)	0 (0.0%)	0 (0.0%)	0 (0.0%)	**1 (0.3%)**
Total	**2 (0.7%)**	**3 (1.1%)**	**1 (0.4%)**	**1 (0.4%)**	**10 (3.7%)**	**2 (0.7%)**	**1 (0.3%)**	**2 (0.7%)**	**3 (1.0%)**	**3 (1.0%)**	**21 (7.3%)**

The bold was just used to track entries

In contrast, several polyresistant strains were detected among HIV positive TB patients. In Nyamira county, INH + KAN; in Homabay county INH + GFX and INH + AMK; and in Kakamega county INH + GFX phenotypes were reported among HIV positive TB patients. In Bungoma, Migori, Kisii, Vihiga, Busia, Kisumu and Siaya there were no polyresistant bacilli detected from among the samples analyzed among HIV positive TB patients (Table 5).

## Discussion

Equal distribution of TB patients among HIV negative and positive sampled in the study area could be attributed to high prevalence of HIV in Western Kenya counties [25]. This is a departure from the national wide survey which indicated high TB prevalence among HIV negative than HIV positive [11]. HIV/AIDS is one of the predisposing factors to tuberculosis infection [26, 27]. Elevated tuberculosis incidences in males than in females is in agreement with finding of Enos et al. [11]. This could be linked to socioeconomic activities; males are the expected to the primary household wage earners (as home bread winners) as dictated by the African culture, and in some cases are forced to work in unhealthy environments. It could also be probable that the high incidence is as a result of increase health care seeking behavior reported in females than in males [28].

Tuberculosis infection has also been associated with social aspects. Social risk factors including poor housing, low income and low education levels as identified in most youths living in Western Kenya counties could be among main drivers to increased tuberculosis incidences among the age groups of 18–35 years old. This age group consist of many youths who lack economic and social stability which predisposes them to increased risks of TB infection [11, 29–31].

Noncompliance to tuberculosis medication regimes could be the main contributor to first and second line antituberculosis drug resistance. Patients in continuation phase of tuberculosis treatment might develop improved signs and symptoms of the disease and therefore think they are cured leading to careless and non-compliance in taking medication. Poor knowledge of tuberculosis and antituberculosis therapy could also contribute to non-compliance leading to increased antituberculosis drug resistance among TB patients in Western Kenya. From the study, 26% of the participants had informal education and 44% had achieved only a primary education level giving a combined total of 70% low education cohort; these findings provide a pointer of how significant education is on adherence to medication and hence increased incidence of antitubercule drug resistance which is in agreement with the finding of [32].

Increased local and international travels which contributes to residents being out of their home towns could be another driver of drugs resistance. It this study it was observed that almost all the participants are involved in local travels with the region, country and far fewer in international travels within East Africa and outside, respectively. Such travels out of home towns had been identified by Obwoge et al., [32] as one of the risk factors to non-adherence to treatment leading to increased tuberculosis drug resistance [33]. Increased international border activities and business engagements living within border counties of Migori, Kisumu, Siaya and Bungoma could be accelerating the risk of drug resistance due to cross border transmission of both drug sensitive and resistant strains. This could be largely because different countries within East Africa could be having Tuberculosis preventive and treatment policies leading to variation in case management.

Additionally, misuse of antimicrobial drugs could also be contributing to increased resistance patterns in Western Kenya. This is in line with the report of Owiti et al. [34], which observed that antimicrobial drug misuse has largely contributed to antimicrobial resistance which has been a major threat to prevention and treatment of a range of pathogens including *Mycobacterium tuberculosis* bacteria. Antimicrobial resistance has been declared a global public health threat and could be one of the drivers to antituberculosis resistance in both first- and second-line anti-tuberculosis drugs as also observed by [33].

Improved healthcare seeking behavior among HIV-TB coinfected patents occasioned by integration of TB and HIV services in primary health care could have contributed to reduced antituberculosis drug resistant in HIV positive TB patients as compared to HIV negative TB patients. The integration of HIV and TB prevention and treatment services has seen increased uptake of Clotrimazole prevention therapy (CPT) and Antiretroviral therapy (ART) among HIV-TB coinfected patients in Kenya [35].

## Conclusion

Drug resistant *M. tuberculosis* strains prevalence is still high among HIV negative and positive patients in Western Kenya with the most affected being HIV negative TB patients. It is therefore probable that the existing control measures are not adequate to control transmission of drug resistant strains. Further, miss diagnosis or delayed diagnosis of TB patients could be contributing to the emergence of *M. tuberculosis* drug polyresistant strains.

## Recommendation

Based on the result of this study, regular TB drug resistance surveillance should be conducted to ensure targeted interventions aimed at controlling increased transmission of the tuberculosis drug resistant strains among HIV/AIDS and HIV negative patients. There is also need for improved drug resistant infection control measures, timely and rapid diagnosis and enhanced and active screening strategies of tuberculosis among suspected TB patients need to be put in place. Further, studies using a larger patient cohort and from counties across the country would shed much needed insights on the true national prevalence of different variants of *M. tuberculosis* drug resistance.

## Data Availability

The data that support the findings of this study are available from Division of Tuberculosis, Leprosy and Lung Diseases, Ministry of Health and Research Division Jomo Kenyatta University of Agriculture and Technology, Kenya but restrictions apply to the availability of these data, which were used under license for the current study, and so are not publicly available. Data are however available from the authors upon reasonable request and with permission of the Head of Division Tuberculosis, Leprosy and Lung Diseases Kenya and Research Division of Jomo Kenyatta University of Agriculture and Technology.
